# Cancer-Testis Gene Biomarkers Discovered in Colon Cancer Patients

**DOI:** 10.3390/genes13050807

**Published:** 2022-05-01

**Authors:** Mikhlid H. Almutairi, Turki M. Alrubie, Abdullah M. Alamri, Bader O. Almutairi, Abdulwahed F. Alrefaei, Maha M. Arafah, Mohammad Alanazi, Abdelhabib Semlali

**Affiliations:** 1 Zoology Department, College of Science, King Saud University, Riyadh 11451, Saudi Arabia; 442106519@student.ksu.edu.sa (T.M.A.); bomotairi@ksu.edu.sa (B.O.A.); afrefaei@ksu.edu.sa (A.F.A.); 2 Genome Research Chair, Department of Biochemistry, College of Science, King Saud University, Riyadh 11451, Saudi Arabia; abdullah@ksu.edu.sa (A.M.A.); msanazi@ksu.edu.sa (M.A.); 3 Pathology Department, College of Medicine, King Saud University, Riyadh 11461, Saudi Arabia; marafah@ksu.edu.sa; 4 Groupe de Recherche en Écologie Buccale, Faculté de Médecine Dentaire, Université Laval, 2420 Rue de la Terrasse, Local 1758, Quebec City, QC G1V 0A6, Canada; abdelhabib.semlali@greb.ulaval.ca

**Keywords:** cancer-testis genes, biomarker, gene expression, colon cancer

## Abstract

In Saudi Arabia, colon cancer (CC) is the most prevalent cancer in men and the third most common cancer in women. Rather than being detected through screening programs, most CC cases are diagnosed mainly during clinical exams. Because of the slow growth of CC and its ability to be treated at an early stage, screening for CC can reduce the incidence of death and mortality. Consequently, there is an urgent need to identify a potential new cancer-specific biomarker for detecting early illness. Much research has been conducted on distinct antigen classes as potential new cancer-specific biomarkers for the early identification of malignancy. The cancer-testis antigens (CTAs) are one such category of antigens, with protein presence largely normally confined to human germ line cells in the testis and aberrantly produced in some cancer cells. CTAs are potentially valuable for use as cancer biomarkers and in cancer therapeutics due to their distinctive expression pattern. The aim of this current study was to identify potential cancer-testis (CT) gene biomarkers in Saudi Arabian CC patients. In this study, a total of 20 matching CC and normal colon (NC) tissues were obtained from the Saudi population. Any genes that showed expression in CC tissues but not in matching NC tissues were subsequently verified for mRNA expression in eight breast and eight leukemia malignancies using RT-PCR to determine the specificity of any CC biomarkers. *CTAG1A, SPZ1, LYZL6, SCP2D1, TEX33,* and *TKTL2* genes were expressed in varying numbers of CC tissues compared to no measurable expressions in all NC tissue specimens, making these genes suitable potential candidates for CC markers. The most frequently expressed CT genes in CC patients were *CTAG1A* (35%) and *SCP2D1* (35%), followed by *TKTL2* (25%), *SPZ1* (20%), *LYZL6* (15%), and *TEX33* (5%). The *LYZL6* gene shows a weak RT-PCR product in 25% of breast cancer (BC) patients but not in leukemia patients. The *SCP2D1* gene appears to display expression in all leukemia patients but not in the BC patients. *TKTL2* expression was also observed in 50% of leukemia samples but not in the BC samples. More experiments at the protein level and with a larger cohort of patients are required to evaluate this finding.

## 1. Introduction

Colon cancer (CC) is a major cause of death worldwide, and it is expected to rise by 60% by 2030 [1]. CC incidence and mortality rates cover an approximately ten-fold range between nations, with the greatest rates observed in wealthier countries, where they have remained relatively steady. However, rates are fast rising in developing countries [1]; despite the fact that colon screening programs have lowered incidence rates in various regions of the world, death rates are still growing in several areas [2,3]. CC is the most frequent type of cancer in males in Saudi Arabia and the third most common type in women [4,5]. In Saudi Arabia, the majority of CC cases are discovered during clinical examinations rather than through screening programs. Screening for CC can minimize the frequency of death and mortality in this illness due to the slow progression of CC and enable the possibility of treatment if discovered at an early stage. As a result, a non-invasive biomarker for early illness identification might be beneficial [6].

CC tumorigenesis is a multistep process including mutations in oncogenes and tumor suppressor genes that leads to the progressive change of the normal colorectal epithelium to adenoma, invasive tumor, and metastatic tumor [7]. The tendency to CC has been linked to a number of risk factors, including ethnicity, environment, and genetics [8].

Early detection and successful cancer treatment remain significant clinical challenges for cancer treatment. As a result, there is a pressing need to discover new tumor-associated molecules that might be used to generate new clinical diagnostics and therapeutic targets for various malignancies [9]. Much research has been conducted on distinct antigen classes as potential new cancer-specific biomarkers for the early identification of malignancy. The cancer-testis antigens (CTAs) are one such category of antigens, with a protein presence largely confined to human germ line cells of the testis and cancer cells [10]. CTAs are potentially valuable for use as cancer biomarkers and cancer therapeutics due to their distinctive, cancer-specific expression pattern [10,11].

Feichtinger et al. (2012) and Sammut et al. (2014) reported a novel set of cancer-testis (CT) genes. These genes were initially discovered using in silico approaches, followed by experimental confirmation [12,13]. The overall goal of the current study was to identify potential CT gene biomarkers in Saudi Arabian CC patients using this published gene set. Twenty-one CT genes have been reported to be expressed in CC cells [12,13]. These genes include *ACTRT1, CCER1, SCP2D1, TEX33, NUTM1, ODF4*, and *TEX19* [12]. *ACTL9, ADAM2, ASB17, C16orf78, CCDC83, LYZL6, PDHA2, PPP3R2, PRPS1L1, SPZ1,* and *ZSWIM2* [13]. Additionally, *C10orf82, CTAG1A,* and *TKTL2* were selected randomly from CTA databases (http://www.cta.lncc.br/index.php, accessed on 1 March 2021). The genes were chosen for two reasons: (1) their expression in CC tissue or cell lines, and (2) their link to malignancy in most of these genes [12,13,14]. Any genes that showed expression in CC tissues but not in matching normal colon (NC) tissues were subsequently verified for mRNA expression in breast and leukemia malignancies using RT-PCR to determine the specificity of any CC biomarkers.

## 2. Materials and Methods

### 2.1. Ethical Approval and Collection of Samples

A total of 36 patients were enrolled in the study, including 20 matching colon cancer (CC) and normal colon (NC) tissues (they are from the same patient), 8 breast cancer (BC) patients, and 8 leukemia patients. CC, NC, and leukemia samples were taken from Saudi male patients, while BC samples were taken from Saudi female patients. All patients were recruited from the King Khalid University Hospital in Riyadh during the years 2019 to 2021. Patients were monitored, diagnosed, and clinical data were gathered using conventional clinical, endoscopic, radiological, and histological criteria confirming adenocarcinoma and, therefore, their eligibility to participate in this study by a panel of surgeons and pathologists. Fresh tissue CC specimens and matched adjacent NC tissues were collected in separate sterile tubes containing RNA*later* stabilization solution (Thermo Fisher; 76106, Foster City, CA, USA) to protect and stabilize RNA.

The Al-Imam Muhammad Ibn Saud Islamic University Ethics Committee accepted the current study, which has the IRB number HAPO-01-R-011 (Project number: 56-2020). Each participant signed a written informed consent form and filled out a survey. Participants were invited to fill out a self-administered questionnaire that asked about their age, family history of cancer, personal medical history, and social behavior such as smoking habits and alcohol consumption. After obtaining informed permission in compliance with the Ethics Committee requirements at Al-Imam Muhammad Ibn Saud Islamic University, clinical information was acquired from each participant.

### 2.2. RNA Extraction from Tissues and Whole Blood

Total RNA was extracted from about 50 mg of CC, NC, and BC samples using the All-Prep DNA/RNA Mini Kit (Qiagen; 80204, Hilden, Germany) according to the manufacturer’s recommendations. In leukemia samples, the total RNA was extracted from about 1.5 mL of whole blood using the QIAamp RNA Blood Mini Kit (Qiagen; 52304, Hilden, Germany) according to the manufacturer’s recommendations. The concentration, purity, and quality of the isolated RNAs were measured using the Nano-Drop8000 spectrophotometer (Thermo Fisher Scientific, Waltham, MA, USA).

### 2.3. cDNA Preparation

Using a High-Capacity cDNA Reverse Transcription Kit (Applied Biosystems; 4368814, Warrington, PA, USA), 1 μg of total RNA from each sample was reverse transcribed into cDNA according to the manufacturer’s instructions. After that, the cDNA was diluted to 1:9 and kept at −20 °C until required.

### 2.4. RT-PCR and Agarose Gel Electrophoresis

For RT-PCR, 0.8 μL of diluted cDNA (85 ng), 0.8 μL of each primer (10 pmol), and 10 μL of BioMix Red (BioLine; BIO-25006, London, UK) were combined with distilled water to make a final volume of 20 μL.

Pre-denaturation hold times of 5 minutes at 96 °C were followed by 35 cycles of denaturing at 96 °C for 30 s, annealing temperature as stated in Table 1 for 30 s, and extension at 72 °C for 30 s/kb, followed by a final extension step of 5 min at 72 °C. 1× TBE buffer was used to run PCR products on 1.5% agarose gels that were stained with 0.5 g/mL ethidium bromide. The quality of normal and cancer cDNA samples was checked by amplification of the housekeeping gene *ACTB*. For the size assessment of the PCR products, 3 μL of 100 bp DNA marker (NEB; N0467, London, UK) was loaded.

### 2.5. Primer Design for RT-PCR

Specific gene sequences are accessible in the National Center for Biotechnology Information’s (http://www.ncbi.nlm.nih.gov/, accessed on 1 March 2021) databases. To avoid false positives due to possible genomic DNA contamination, intron-spanning primers were constructed for each gene. The specific primer for each gene was created using Primer-BLAST software (https://www.ncbi.nlm.nih.gov/tools/primer-blast/, accessed on 1 March 2021). All primers used in this study were synthesized commercially (Macrogen Inc., Seoul, South Korea) and diluted to a final concentration of 10 pmol using sterile distilled water. The primer sequences for each gene and their predicted size are listed in Table 1.

### 2.6. Purification and Sequencing of RT-PCR Products

The PCR products were separated on a 1% agarose gel electrophoresis. The amplified products were then purified using the Roche Applied Science High Pure PCR Product Purification Kit (Roche; 11732668001, Darmstadt, Germany). Then, 10 ng/μL of DNA in a total volume of 10 μL was placed in a clean 1.5 mL Eppendorf tube, and 5 pmol/μL forward and/or reverse primers in a total volume of 10 μL were transferred to additional tubes. DNA sequencing was undertaken by Microgen. To compare a query sequence with the NCBI databases of sequences, the resulting sequencing of each product was submitted to the Basic Local Alignment Search Tool (BLAST) website (https://blast.ncbi.nlm.nih.gov/Blast.cgi, accessed on 1 March 2021) and the EMBL European Bioinformatics Institute website (https://www.ebi.ac.uk/, accessed on 1 March 2021) was used to predict the sequence of PCR product.

### 2.7. Primer Design for Real Time Quantitative PCR (qRT-PCR)

All the primers were manually designed with an amplicon size of 180 bp for efficient amplification in qRT-PCR. Each primer had 20 nucleotides in length and included 50–55 percent G/C to avoid the projected internal secondary structure. To prevent primer-dimer formation, the forward and reverse primers had no substantial complementarity at the 3’ ends and had equal melting temperatures. A BLAST search was used to examine primers to ensure specificity. Primers were synthesized commercially (Macrogen Inc., South Korea), and their sequences are enlisted in Table 2. Stock primers were diluted to a final concentration of 10 pmol using sterile distilled water.

### 2.8. PCR Setup for qRT-PCR

The qRT-PCR experiments were set up using the iTaq Universal SYBR Green Supermix (Bio-Rad; 1725120, Hercules, CA, USA) according to the manufacturer’s instructions. Then, 5 μL of SYBR Green Supermix, 2 μL of cDNA (200 ng), 0.25 μL from each primer, and finally water was added to adjust the volume to 10 μL in a 96-well plate. Samples were duplicated three times and amplified using a pre-denaturation phase of 30 s at 95 °C, followed by 40 cycles of 15 s at 95 °C, 30 s of primer annealing at 60 °C, and 15 s of extension at 95 °C. After the 40 cycles were completed, a melting curve analysis was performed. For normalization of the qRT-PCR findings, *GAPDH* was used as a positive control. A QuantStudioTM 7 Flex Real-time PCR System was used to perform qRT-PCR (Applied Biosystems, Hercules, CA, USA).

### 2.9. Statistical Analysis

An unpaired Student t-test was used to analyse the differences between two groups (NC and CC tissues) of *ACTL9, PDHA2, SCP2D1*, and *TKTL2* expressions. Statistical significance was determined for all *p* values (* *p* < 0.05, ** *p* < 0.01).

## 3. Results

### 3.1. Clinical Data on the Studied Subjects

The late diagnosis of CC is one of the most significant causes of increased mortality in Saudi Arabia, as it is more difficult to treat at later stages. Consequently, studying CT gene expressions in multiple patients with CC could help in the detection of cancer in the early stages (i.e., a cancer biomarker) and thereby increasing the possibility of treatment.

The general demographic and clinical characteristics of the study participants are shown in Table 3. A total of 36 patients were tested, including 20 samples of NC and CC, eight samples of BC, and eight samples of leukemia patients. The mean age of the CC, BC, and leukemia patients, according to our study on the general demographic aspects of the donors, was 60 (ranged from 24 to 83), 54 (ranged from 46 to 74), and 52 (ranged from 32 to 61) years, respectively. Thirty-five percent of the CC patients were under the age of 60, while sixty-five percent were over 60 years old. Overall, 62.5% of BC and leukemia patients were under the age of 54, and 52, respectively, while 37.5% were above the age of 54, and 52 years old, respectively. Other clinical characteristics of these individuals, including tumor grade, leukemia type, and estrogen receptor and progesterone receptor status, are presented in Table 3, Table 4 and Table 5.

### 3.2. Expression Profile of The Selected Genes in CC Tissues and Matching NC Tissues

mRNA levels for the genes indicated in Table 1 were validated by RT-PCR analysis with a variety of RNAs from 20 human colon normal tissues to evaluate their testis-specificity. The primer for each gene was validated using testis cDNA generated from human testes’ total RNA (Thermo Fisher Scientific, Waltham, MA, USA; AM7972). The expression of *ACTB* served as a positive control to validate the quality of the cDNA. A triplicate PCR was conducted individually for each gene.

**Figure 1:**
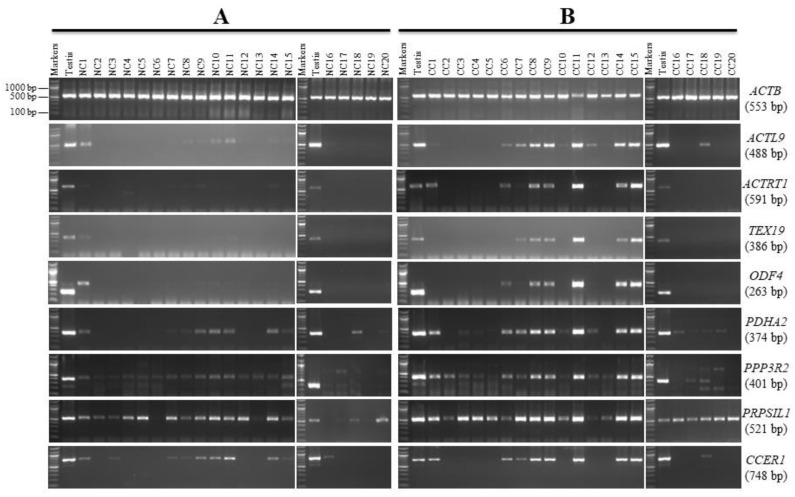
RT-PCR expression profiles for the *ACTL9, ACTRT1, TEX19, ODF4, PDHA2, PPP3R2, PRPSIL1,* and *CCER1* genes in matching normal colon (NC) and colon cancer (CC) tissues. Agarose gels display the RT-PCR analysis for the *ACTL9, ACTRT1, TEX19, ODF4, PDHA2, PPP3R2, PRPSIL1,* and *CCER1* genes. cDNAs were synthesized from the total RNA from 20 NC tissues (**A**) and CC tissues (**B**). *ACTB* expression was used as a positive control for the cDNA samples. Testis cDNA was used to test the primer for each gene. The expected product size of each gene is presented on the right between brackets.

The RT-PCR screening of the selected genes on the twenty NC tissues indicated that eight genes (*ACTL9, ACTRT1, TEX19, ODF4, PDHA2, PPP3R2, PRPS1L1,* and *CCER1*) showed faint bands in different NC tissues (Figure 1A) and strong bands in CC tissues (Figure 1B).

*ODF4* RT-PCR analysis in the NC and CC tissues showed an unexpectedly large band (about 480 bp) compared to 263 bp in the normal testis. The sequence of this PCR product was related to *ODF4* (Supplementary Table S1), which might point to a different splice variant for the *ODF4* gene.

**Figure 2:**
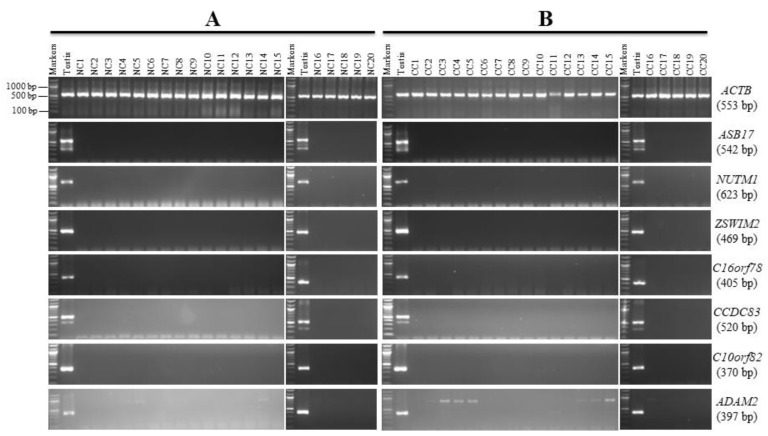
RT-PCR expression profiles for the meiotic restricted genes in matching normal colon (NC) and colon cancer (CC) tissues. Agarose gels display the RT-PCR analysis for the *ASB17, NUTM1, ZSWIM2, C16orf78, CCDC83, C10orf82, and ADAM2* genes. cDNAs were synthesized from the total RNA from 20 NC tissues (**A**) and CC tissues (**B**). *ACTB* expression was used as a positive control for the cDNA samples. Testis cDNA was used to test the primer for each gene. The expected product size of each gene is presented on the right between brackets.

**Figure 3:**
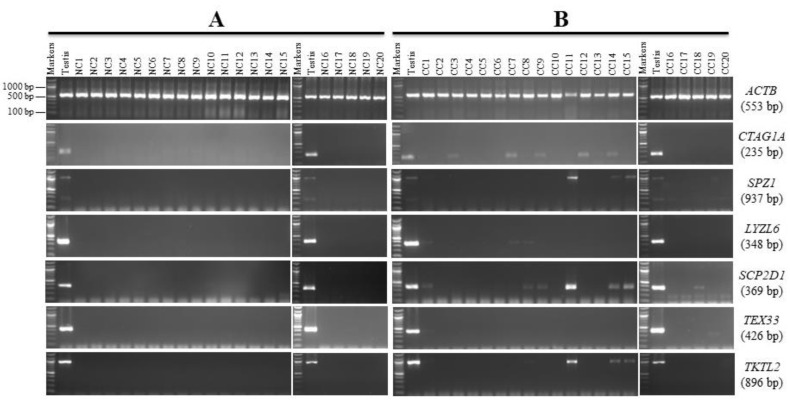
RT-PCR expression profiles for the candidate colon cancer markers in matching normal colon (NC) and colon cancer (CC) tissues. Agarose gels display the RT-PCR analysis for the *CTAG1A, SPZ1, LYZL6, SCP2D1, TEX33,* and *TKTL2* genes. cDNAs were synthesized from the total RNA from 20 NC tissues (**A**) and CC tissues (**B**). *ACTB* expression was used as a positive control for the cDNA samples. Testis cDNA was used to test the primer for each gene. The expected product size of each gene is presented on the right between brackets.

The expression of 13 genes (*ASB17, NUTM1*, *ZSWIM2, C16orf78, CCDC83*, *C10orf82, ADAM2, CTAG1A, SPZ1, LYZL6, SCP2D1, TEX33,* and *TKTL2*) was restricted to the testis in the NC tissue panel (Figure 2A and Figure 3A). Therefore, we further investigated the previous genes by RT-PCR in a range of twenty CC tissues to find any CC markers among those genes. *ASB17, NUTM1, ZSWIM2, C16orf78, CCDC83, C10orf82,* and *ADAM2* were found to have no expression in the CC samples utilized in this investigation; hence, they were designated as testis-specific genes since their expression was limited to the normal testis alone. These seven genes, however, were not ruled out as CC candidates, and additional testing in other CC tissues and/or other tissues from different cancers not included in this study may reveal that they are expressed. The anticipated PCR product size for the *ADAM2* gene in testis cDNA is 397 bp (Table 1); however, the PCR product in the NC tissues and CC tissues showed a larger band of approximately 1000 bp, but this larger band was not related to the *ADAM2* gene (Supplementary Table S1).

*CTAG1A, SPZ1, LYZL6, SCP2D1, TEX33,* and *TKTL2* were expressed in varying numbers of CC tissues compared to no measurable expressions in all NC tissue specimens. PCR products observed from CC samples make these genes suitable potential candidates for CC markers (Figure 3B). The most frequently expressed CT genes in CC were *CTAG1A* (35%) and *SCP2D1* (35%), followed by *TKTL2* (25%), *SPZ1* (20%), *LYZL6* (15%), and *TEX33* (5%) (Figure 3B).

The findings from the DNA sequencing for the genes *C10orf82, CTAG1A, ACTRT1, SPZ1, LYZL6, SCP2D1, TEX33,* and *TKTL2* were compared to the reference sequence in the NCB1 using the BLAST program (http://blast.ncbi.nlm.nih.gov, accessed on 1 March 2021) (Supplementary Table S1). The BLAST software was used to blast the sequences retrieved from the sequencing.

### 3.3. Studying the Specificity of the CC Biomarkers Identified in the Selected Genes

To determine their specificity, six of the CC biomarkers identified in Figure 3: *CTAG1A, LYZL6, SCP2D1, TEX33, TKTL2*, and *SPZ1* were screened for expression in five samples of chronic myeloid leukemia (CML), three samples of chronic lymphoblastic leukemia (CLL), and eight samples of BC isolated from the Saudi population.

**Figure 4:**
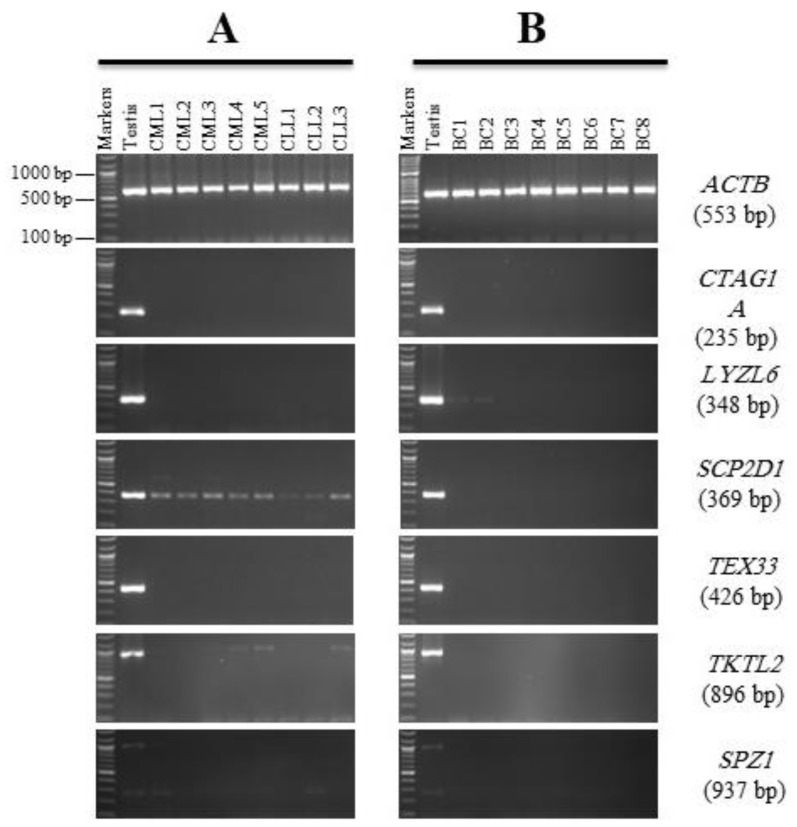
RT-PCR expression profiles for the candidate colon cancer markers in leukemia and breast cancer (BC) tissues. Agarose gels display the RT-PCR analysis for the *CTAG1A, LYZL6, SCP2D1, TEX33, TKTL2,* and *SPZ1* genes. (**A**) cDNAs were synthesized from the total RNA from eight leukemia cancer (chronic myeloid leukemia (CML), and chronic lymphoblastic leukemia (CLL)). (**B**) cDNAs were synthesized from the total RNA from BC tissues. *ACTB* expression was used as a positive control for the cDNA samples. Testis cDNA was used to test the primer for each gene. The expected product size of each gene is presented on the right between brackets.

According to the expression profile seen in Figure 3B, *CTAG1A*, *TEX33*, and *SPZ1* were originally classed as CC markers. However, in leukemia and BC tissues, there were no measurable mRNAs (Figure 4A,B).

The *LYZL6* gene shows a weak RT-PCR product in 25% of the BC samples (Figure 4B) but not in CML and CLL samples (Figure 4A). The sequencing results of the purified PCR products ensured the correct target; therefore, it suggests that *LYZL6* could be considered a marker for CC and BC for the Saudi population.

Another gene identified as a CC marker due to its expression profile (Figure 3) is *SCP2D1*. Although the *SCP2D1* gene appears to display an expression in the CC tissues, this mRNA is also presented in all leukemia samples (Figure 4A). However, no expression was detected in any of the BC tissues (Figure 4B). Therefore, *SCP2D1* expression appears to be restricted to the CC, CML, and CLL tissues, as shown in Figure 3B and Figure 4A.

The expression of *TKTL2* was restricted to the testes in the NC tissues, with additional expression also being shown in 25% of the CC tissues (Figure 3B). *TKTL2* expression was also observed from a very weak RT-PCR product in 50% of theleukemia samples (Figure 4A) but not in the BC samples tested in this investigation (Figure 4B). 

### 3.4. Screening of Meiotic Genes in CML, CLL, and BC Tissues

The goal of this screening was to find possible novel CT genes that might be employed as cancer markers and/or therapeutic targets. The *NUTM1, C10orf82, C16orf78, ASB17, ZSWIM2, CCDC83*, and *ADAM2* genes showed no expression in the NC or CC tissues; hence they were designated as testis-restricted following validation. 

**Figure 5:**
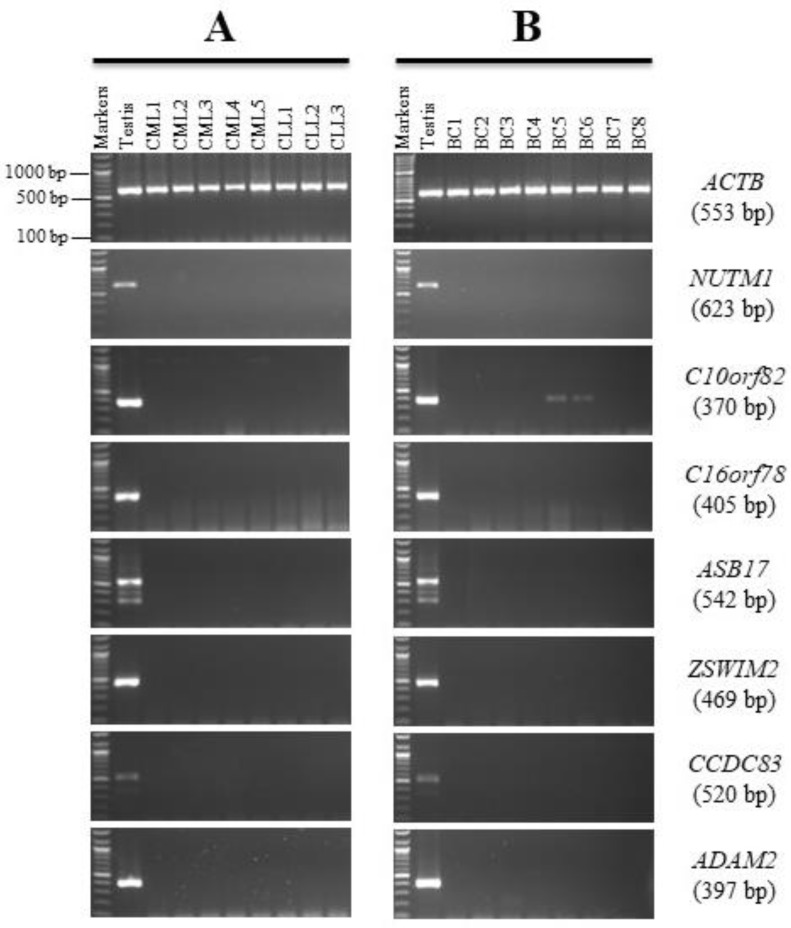
RT-PCR expression profiles for the meiotic restricted genes in leukemia and breast cancer (BC) tissues. Agarose gels display the RT-PCR analysis for the *NUTM1, C10orf82, C16orf78, ASB17, ZSWIM2, CCDC83,* and *AMAM2* genes. (**A**) cDNAs were synthesized from the total RNA from eight leukemia cancer (chronic myeloid leukemia (CML), and chronic lymphoblastic leukemia (CLL)). (**B**) cDNAs were synthesized from the total RNA from breast cancer (BC) tissues. *ACTB* expression was used as a positive control for the cDNA samples. Testis cDNA was used to test the primer for each gene. The expected product size of each gene is presented on the right between brackets.

The expression patterns for all six genes were clearly testis-restricted since no expression was detected in any leukemia and/or BC tissues, except for *C10orf82* (Figure 5A,B). The RT-PCR expression profile of *C10orf82* showed a weak band in 25% of the BC tissues. Sequencing of this PCR product showed significant sequence similarity to *C10orf82* (Supplementary Table S1). Therefore, the *C10orf82* gene may be a BC-specific gene worth investigating.

### 3.5. qRT-PCR Analysis of ACTL9, PDHA2, SCP2D1, and TKTL2 Expressions in CC and NC Tissues

**Figure 6:**
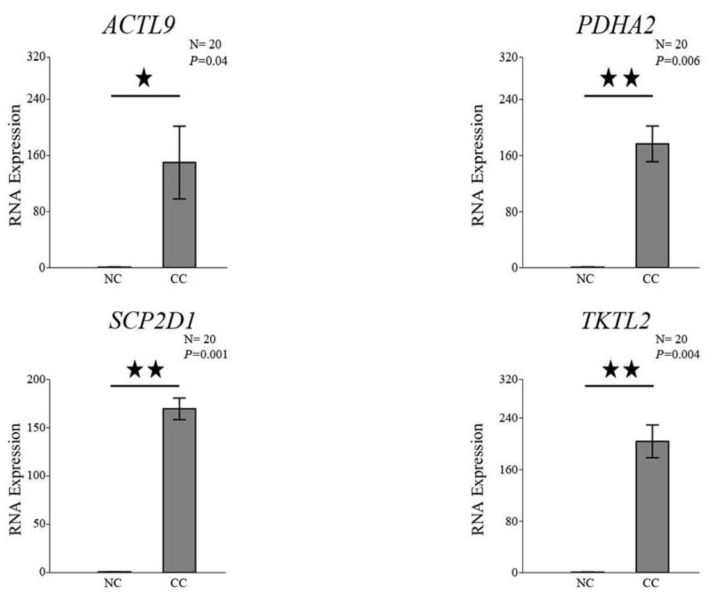
qRT-PCR analysis of *ACTL9, PDHA2, SCP2D1,* and *TKTL2* expressions in colon cancer (CC) and normal colon (NC) tissues. The gene expression data for *ACTL9, PDHA2, SCP2D1*, and *TKTL2* in CC and NC tissues are shown in the bar chart. The *GAPDH* reference gene was used to normalize the expression data. The standard error of the mean for three repetitions is shown by the error bars (★ *p* < 0.05, ★★ *p* < 0.01). N = number of samples.

For further analysis, qRT-PCR was used to measure the levels of *ACTL9, PDHA2, SCP2D1*, and *TKTL2* mRNA in 20 CC and 20 NC tissues. We evaluated the expression of each gene in NC and its match of CC in each sample. Then, the expression of each gene in NC was normalized to one and compared to its match of CC. After this, we took the average and standard error of the expression of each gene in all samples. According to the qRT-PCR data, Figure 6 shows that the expression of *ACTL9, PDHA2, SCP2D1*, and *TKTL2* is greater in the CC tissues than in the NC tissues. As a result, the qRT-PCR results correspond to the RT-PCR results seen previously in Figure 1 and Figure 3.

## 4. Discussion

CTAs are cancer-specific biomarkers with promising prognostic or diagnostic and therapeutic applications. Hoffman and colleagues [15] proposed the current categorization scheme for CT genes which is currently in use. A sub-category of meiosis-specific genes has been ascribed to CT genes based on an in silico pipeline, beginning with probable meiotic genes [12].

In the current study, 21 CT genes were quantified using RT-PCR analysis in 20 CC tissues and compared with their matching NC tissues. According to the expression profiles, these predicted meiosis-specific genes showed elevated expression in different types of CC samples, indicating their potential as biomarkers.

The RT-PCR analysis of *ACTL9, ACTRT1, TEX19, ODF4, PDHA2, PPP3R2, PRPS1L1,* and *CCER1* genes showed PCR product in multiple NC tissues in addition to multiple CC tissues; however, their bands in CC tissues were found to be stronger than the NC tissues. This pattern was also found in a previous study, demonstrating the greater expression of *ACTL9, ACTRT1, PDHA2, PPP3R2, PRPS1L1,* and *CCER1* genes among CC cell lines [12,13]. Similar results were observed for *TEX19* in CC cell lines [12] and bladder cancer [16]. *ODF4* was also upregulated in BC tissues, as compared with their adjacent normal tissues [17], which is similar to our RT-PCR results. qRT-PCR analyses for the above eight genes are required to confirm the RT-PCR results; unfortunately, we only performed analyses for *ACTL9* and *PDHA2* because the majority of CC samples had run out. Interestingly, we found that the qRT-PCR results *ACTL9* and *PDHA2* corresponded to the RT-PCR results, confirming that these genes were overexpressed in CC tissues as compared to NC tissues.

Furthermore, this screen identified six genes as potential novel CT genes. These genes were characterized after validation as CT-restricted genes, including *CTAG1A, SPZ1, LYZL6, SCP2D1, TEX33,* and *TKTL2*. Therefore, all these genes were found to be optimal candidates as CC markers in the Saudi population since they were observed to show expression in different types of CC samples but not in NC tissues. This clustering phenomenon is probably linked to the activation process for CT genes in cancer. For example, demethylation in cancer leads to the activation of CT genes [18]. To identify the gene specificity using BC and leukemia samples, only three genes showed expression among these tissues. These genes are *LYZL6* in BC tissues and *SCP2D1* and *TKTL2* genes in the majority of leukemia tissues. To study CC specificity, BC and leukemia samples are inadequate. The number of patients was relatively small, and this may have affected the statistical results. It was difficult to collect more samples due to traditional restrictions, and participants did not cooperate with the tissue sampling provided; therefore, more samples of BC, leukemia, and other cancers are required.

Previous studies identified the same findings for *CTAG1A*, *SPZ1*, *LYZL6, SCP2D1,* and *TEX33* genes. *CTAG1A* (*NY-ESO-1*) was expressed in patients with colorectal and lung cancers among the Chinese population [19,20]. Due to the sample limitation, an investigation was conducted on the role of specific genes *CTAG1A*, *SPZ1*, and *TEX33* in CC progression. We evaluated mRNA expression levels using an interactive online database OncoDB [21], mainly from TCGA and GTEx [22]. Therefore, a bioinformatic RNA-seq data analysis pipeline was used, adopting standard features recommended by the GDC (https://docs.gdc.cancer.gov/, accessed on 1 March 2021), outcomes with significantly higher expression levels in CC tissues compared to other cancers and normal tissues. Moreover, considerable attention has been focused on the *CTAG1A* gene due to the marked cellular and humoral immune responses it induces [23]. Such responses are often detected in patients with breast [24], gastric [25], esophageal [26], lung [27], and hepatocellular [28] cancer but are more highly expressed in the CC. This response is considered a novel serological biomarker and has a specific immunotherapeutic clinical application method for colorectal cancer [19]. Correspondingly, a significant strong association and higher expression have been revealed in a recent investigation of *SPZ1* using in vitro and in vivo methods, demonstrating that *SPZ1* contributes to tumor progression by inhibiting apoptosis. This is a suggestion that it might be used as a biomarker target for colorectal cancer [29]. The *LYZL6* gene was expressed in a CC cell line [13], while the *SCP2D1* (*C20orf79*) and *TEX33* (*C22orf33*) genes were found to be expressed in diverse types of cancer cell lines, including CC type [12]. Zhao et al. demonstrated that *TKTL1* was more highly expressed in ovarian cancer tissues than in normal tissues [30]. However, no expression was observed in *TKTL2* between all ovarian cancers tested when compared with normal ovarian tissues [30].

In contrast, the *NUTM1, C10orf82, C16orf78, ASB17, ZSWIM2, CCDC83,* and *ADAM2* genes were shown to have a testis-restricted expression pattern in the NC tissues and demonstrated no evidence of RT-PCR expression in the CC tissues. However, these genes were not dismissed from the gene screening because they might be expressed in other cancer types. Therefore, RT-PCR analyses for these seven genes were carried out in different types of BC and leukemia tissues. Interestingly, only the *C10orf82* gene was expressed in BC, as compared to CC and leukemia tissues. This result corresponds with a previous study that showed *C10orf82* expression was closely linked with ovarian cancer patients [31]. *CCDC83-1, CCDC83-2,* and *CCDC83-3* are three variations of the *CCDC83* gene, which contains 11 exons. In this study, we tested *CCDC83* variant 1, and mRNA expression was not found in CC tissues. A previous study identified the same results for *CCDC83-1* but in CC cell lines; however, *CCDC83-2* and *CCDC83-3* mRNA expression was found in various cancer cell lines [32]. The expression of the *ADAM2* gene was not detected in this study among the three types of cancer tissues, which is consistent with another report using tissues from BC samples [33]. The *C16orf78, ASB17,* and *ZSWIM2* gene findings among the Saudi population were discordant with Sammut et al. study that identified their expressions in different CC cell lines [13]. The *NUTM1* gene was expressed in the testis only and was absent in the CC, BC, and leukemia tissues. This result was, therefore, inconsistent with another report that determined its expression profile at a low level in the HCT116 colon cancer cell line [12].

Lastly, our study aims to identify CT gene biomarkers for the early diagnosis of CC that could help screen the potential candidates for CC. However, there are some limitations to the present study. First, the surgical samples numbered only 20, and the results need to be replicated in larger samples. Second, we did not evaluate the protein levels of the candidate CT genes in CC due to a lack of samples.

## 5. Conclusions

In this study, we analyzed the expression profiles of 21 CT genes in CC tissues and their matching NC tissues. We found that *CTAG1A, SPZ1, LYZL6, SCP2D1, TEX33*, and *TKTL2* genes showed mRNA expression in different CC patients but not in adjacent NC tissues. The expression pattern of these genes in CC samples suggests that they may be used as cancer biomarkers for the early diagnosis of CC. However, more experiments at the protein level and with a larger cohort of patients are required to evaluate this finding. In addition, the mechanism responsible for the expression of these genes in tumorigenesis requires further evaluation.

## Figures and Tables

**Table 1:** Primer sequences used in RT-PCR study and their expected product size.

Gene (Official Symbol)	Chromosomal Location	Primer Direction	Primer Sequence (From 5’→3’)	Ta *	Product Size (bp)
*ACTB*	7	Forward	AGAAAATCTGGCACCACACC	58	553
		Reverse	AGGAAGGAAGGCTGGAAGAG		
*ACTL9*	19	Forward	CAGTCGGTGCTGTCTGTCTA	60	488
		Reverse	CCGCAGAGAAGCACGTTTTG		
*ADAM2*	8	Forward	GTCTTGTTTCTGCTCAGCGG	60	397
		Reverse	AGCCAACTGAAGACTCCAGG		
*ASB17*	1	Forward	GTGGGGATATCACTGTTACG	58	542
		Reverse	GCACTCTGGAACATAGTACC		
*C10orf82*	10	Forward	CTGCCAAGGAATGTCCAAG	60	370
		Reverse	ATGTGCCTTCTTGGCCCTCT		
*C16orf78*	16	Forward	CAGGGGAAGAAGAAACAAGC	58	405
		Reverse	GTCTCTTATGAAGGTTGCCC		
*CCDC83*	11	Forward	GAGAGGATGTTGAAGAAGCG	58	520
		Reverse	CTGGGTATCTTGAGATCCAC		
*LYZL6*	17	Forward	GGCGCTACTCATCTATTTGG	58	348
		Reverse	CCGGACACAATCCTTTTTGC		
*CTAG1A*	X	Forward	CCTGCTTGAGTTCTACCTCG	60	235
		Reverse	CTGCGTGATCCACATCAACA		
*PDHA2*	4	Forward	CGAGTTGCCCAGAAATCAGC	60	374
		Reverse	AGCTCTGCGAGAATGGATCG		
*PPP3R2*	9	Forward	GGGCAGGAGGTTTAAGAAGT	58	401
		Reverse	CCACAGCACTGAATTCCTCA		
*PRPS1L1*	7	Forward	GTCTACATCGTTCAGAGTGG	58	521
		Reverse	CAAGTGTCTGCCATGTCATC		
*NUTM1*	15	Forward	CACCACCAGTTGCTCAACTG	60	623
		Reverse	CTCCTTCACAGCTTCTGGTG		
*TEX19*	17	Forward	GCTTCAACATGGAGATCAGC	58	386
		Reverse	GAAGCTCCTCAAATCTCCAG		
*SPZ1*	5	Forward	CTGCTAAGTCAGCTGAGATG	58	937
		Reverse	GAATAGGTGTCATGGCTCAG		
*TKTL2*	4	Forward	AGGTACTGCATGTGGAATGG	58	896
		Reverse	CATCTTCTCCAGTGGATACC		
*ZSWIM2*	2	Forward	GACAAACACCTTGGGATTCC	58	469
		Reverse	GGCATGAATTGCACTTGTGG		
*ODF4*	17	Forward	CCTTCATCTTCTCCACCCTC	60	263
		Reverse	GGTGTCTGTGATCGTCTGTG		
*CCER1*	12	Forward	CAGCGTACAATAGACCGCAC	60	748
		Reverse	CACACCTCCTGGTCATACTC		
*ACTRT1*	X	Forward	GGGATGACATGGAGAAACTC	58	591
		Reverse	CCATTTTTGAGAGTCCTGGG		
*SCP2D1*	20	Forward	CAGTTCGAGGTTCTGGGTTC	60	369
		Reverse	GCTAAGCAGAACCTTGCCAC		
*TEX33*	22	Forward	GATCCTCCTCGAGAGAGAAC	60	426
		Reverse	GCCAGTGTTCTAAGTCCCTC		

Ta *—Annealing temperature for each gene.

**Table 2:** Primer sequences for qRT-PCR and their expected amplicon size.

Gene (Official Symbol)	Primer Direction	Primer Sequence (From 5’→3’)	Product Size (bp)
*GAPDH*	Forward	GGGAAGCTTGTCATCAATGG	173
	Reverse	GAGATGATGACCCTTTTGGC	
*ACTL9*	Forward	CAAGGAGCTGTTCCAGTGTC	153
	Reverse	CCGCAGAGAAGCACGTTTTG	
*PDHA2*	Forward	GATGGTCAGGAAGCTTGTTG	133
	Reverse	TCAGCTCTGCGAGAATGGAT	
*TKTL2*	Forward	CATGGTAAGTGTGGCACTAG	149
	Reverse	CACAGTGGGAACCAATAAGG	
*SCP2D1*	Forward	CCAGCAGACACTGTCTTTAC	129
	Reverse	CTTCCAGCTAAGCAGAACCT	

**Table 3:** General clinical parameters of the study participants.

Variables	Colon Cancer N (%)	Normal Colon N (%)	Breast Cancer N (%)	Leukemia N (%)
Participants	20 (100%)	20 (100%)	8 (100%)	8 (100%)
Sex				
Males	20 (100%)	20 (100%)	-----	8 (100%)
Females	-----	-----	8 (100%)	-----
Mean age (min–max)	60 (24–83)		54 (46–74)	52 (32–61)
Below 60	7 (35%)	7 (35%)	-----	-----
Above 60	13 (65%)	13 (65%)	-----	-----
Below 54	-----	-----	5 (62.5%)	-----
Above 54	-----	-----	3 (37.5%)	-----
Below 52	-----	-----	-----	5 (62.5%)
Above 52	-----	-----	-----	3 (37.5%)
Estrogen Receptor (ER)				
ER+	-----	-----	1 (12.5%)	-----
ER-	-----	-----	7 (87.5%)	-----
Progesterone Receptor (PR)				
PR+	-----	-----	2 (25%)	-----
PR-	-----	-----	6 (75%)	-----
Type of Leukemia				
Chronic myeloid	-----	-----	-----	5 (62.5%)
Chronic lymphoblastic	-----	-----	-----	3 (37.5%)

**Table 4:** Colon cancer (CC) patients’ ages and their cancer grades.

Variable	CC Patients																			
Sample	1	2	3	4	5	6	7	8	9	10	11	12	13	14	15	16	17	18	19	20
Ages	79	49	63	38	79	73	54	24	69	61	38	69	65	47	55	83	61	73	48	78
Cancer grade	II	I	III	II	IIII	II	II	II	II	II	III	II	II	III	III	II	II	II	II	II

**Table 5:** Breast cancer (BC) and leukemia patients’ ages and their cancer grades.

Variable	BC Patients								Leukemia Patients							
Sample	1	2	3	4	5	6	7	8	1	2	3	4	5	6	7	8
Ages	49	48	46	52	46	67	74	55	51	61	51	58	32	51	61	51
Cancer grade	II	III	IIII	III	II	II	II	I	---	---	---	---	---	---	---	---

## Data Availability

All data generated or analyzed during this study are included in this published article.

## Supplementary Materials

The following supporting information can be downloaded at: https://www.mdpi.com/article/10.3390/genes13050807/s1, Table S1: Summary of the sequencing results for the desired genes.

## Abbreviations

CC	Colon cancer
CTAs	Cancer-testis antigens
CT	Cancer-testis
NC	Normal colon
BC	Breast cancer
CML	Chronic myeloid leukemia
CLL	Chronic lymphoblastic leukemia
mRNA	messenger RNA
RNA	Ribonucleic acid
mg	Milligram
cDNA	Complementary DNA
PCR	Polymerase chain reaction
RT-PCR	Reverse transcription PCR
qRT-PCR	quantitative, real-time PCR
bp	Base pair
kb	Kilobase
BLAST	Basic Local Alignment Search Tool
Ta	Annealing temperature
pmol	picomole
μg	Microgram
ng	Nanogram
mL	Milliliter
μL	Microliter

## References

[1] Arnold M., Sierra M.S., Laversanne M., Soerjomataram I., Jemal A., Bray F. (2017). Global patterns and trends in colorectal cancer incidence and mortality. Gut.

[2] Dekker E., Tanis P.J., Vleugels J.L.A., Kasi P.M., Wallace M.B. (2019). Colorectal cancer. Lancet.

[3] Issa I.A., Noureddine M. (2017). Colorectal cancer screening: An updated review of the available options. World J. Gastroenterol..

[4] Althubiti M.A., Nour Eldein M.M. (2018). Trends in the incidence and mortality of cancer in Saudi Arabia. Saudi Med. J..

[5] Herzallah H.K., Antonisamy B.R., Shafee M.H., Al-Otaibi S.T. (2019). Temporal trends in the incidence and demographics of cancers, communicable diseases, and non-communicable diseases in Saudi Arabia over the last decade. Saudi Med. J..

[6] Kanojia D., Garg M., Gupta S., Gupta A., Suri A. (2011). Sperm-associated antigen 9 is a novel biomarker for colorectal cancer and is involved in tumor growth and tumorigenicity. Am. J. Pathol..

[7] Fearon E.R., Vogelstein B. (1990). A genetic model for colorectal tumorigenesis. Cell.

[8] Sameer A.S. (2013). Colorectal cancer: Molecular mutations and polymorphisms. Front. Oncol..

[9] Suri A., Jagadish N., Saini S., Gupta N. (2015). Targeting cancer testis antigens for biomarkers and immunotherapy in colorectal cancer: Current status and challenges. World J. Gastrointest. Oncol..

[10] Whitehurst A.W. (2014). Cause and consequence of cancer/testis antigen activation in cancer. Annu. Rev. Pharmacol. Toxicol..

[11] Krishnadas D.K., Bai F., Lucas K.G. (2013). Cancer testis antigen and immunotherapy. Immunotargets Ther..

[12] Feichtinger J., Aldeailej I., Anderson R., Almutairi M., Almatrafi A., Alsiwiehri N., Griffiths K., Stuart N., Wakeman J.A., Larcombe L. (2012). Meta-analysis of clinical data using human meiotic genes identifies a novel cohort of highly restricted cancer-specific marker genes. Oncotarget.

[13] Sammut S.J., Feichtinger J., Stuart N., Wakeman J.A., Larcombe L., McFarlane R.J. (2014). A novel cohort of cancer-testis biomarker genes revealed through meta-analysis of clinical data sets. Oncoscience.

[14] Almeida L.G., Sakabe N.J., Deoliveira A.R., Silva M.C.C., Mundstein A.S., Cohen T., Chen Y., Chua R., Gurung S., Gnjatic S. (2009). CTdatabase: A knowledge-base of high-throughput and curated data on cancer-testis antigens. Nucleic Acids Res..

[15] Hofmann O., Caballero O.L., Stevenson B.J., Chen Y.T., Cohen T., Chua R., Maher C.A., Panji S., Schaefer U., Kruger A. (2008). Genome-wide analysis of cancer/testis gene expression. Proc. Natl. Acad. Sci. USA.

[16] Zhong J., Chen Y., Liao X., Li J., Wang H., Wu C., Zou X., Yang G., Shi J., Luo L. (2016). Testis expressed 19 is a novel cancer-testis antigen expressed in bladder cancer. Tumour Biol..

[17] Kazemi-Oula G., Ghafouri-Fard S., Mobasheri M.B., Geranpayeh L., Modarressi M.H. (2015). Upregulation of RHOXF2 and ODF4 Expression in Breast Cancer Tissues. Cell J..

[18] Almatrafi A., Feichtinger J., Vernon E.G., Escobar N.G., Wakeman J.A., Larcombe L.D., McFarlane R.J. (2014). Identification of a class of human cancer germline genes with transcriptional silencing refractory to the hypomethylating drug 5-aza-2′-deoxycytidine. Oncoscience.

[19] Li Y., Song R., Li X., Xu F. (2017). Expression and immunogenicity of NY-ESO-1 in colorectal cancer. Exp. Ther. Med..

[20] Jin S., Cao S., Li J., Meng Q., Wang C., Yao L., Lang Y., Cao J., Shen J., Pan B. (2018). Cancer/testis antigens (CTAs) expression in resected lung cancer. Onco Targets Ther..

[21] Tang G., Cho M., Wang X. (2022). OncoDB: An interactive online database for analysis of gene expression and viral infection in cancer. Nucleic Acids Res..

[22] Consortium G.T. (2015). Human genomics. The Genotype-Tissue Expression (GTEx) pilot analysis: Multitissue gene regulation in humans. Science.

[23] Jager E., Chen Y.T., Drijfhout J.W., Karbach J., Ringhoffer M., Jäger D., Arand M., Wada H., Noguchi Y., Stockert E. (1998). Simultaneous humoral and cellular immune response against cancer-testis antigen NY-ESO-1: Definition of human histocompatibility leukocyte antigen (HLA)-A2-binding peptide epitopes. J. Exp. Med..

[24] Yang Z., Chevolot Y., Géhin T., Solassol J., Mange A., Souteyrand E., Laurenceau E. (2013). Improvement of protein immobilization for the elaboration of tumor-associated antigen microarrays: Application to the sensitive and specific detection of tumor markers from breast cancer sera. Biosens. Bioelectron..

[25] Fujiwara S., Wada H., Kawada J., Kawabata R., Takahashi T., Fujita J., Hirao T., Shibata K., Makari Y., Iijima S. (2013). NY-ESO-1 antibody as a novel tumour marker of gastric cancer. Br. J. Cancer.

[26] Xu Y.W., Peng Y.H., Chen B., Wu Z.Y., Wu J.Y., Shen J.H., Zheng C.P., Wang S.H., Guo H.P., Li E.M. (2014). Autoantibodies as potential biomarkers for the early detection of esophageal squamous cell carcinoma. Am. J. Gastroenterol..

[27] Shan Q., Lou X., Xiao T., Zhang J., Sun H., Gao Y., Cheng S., Wu L., Xu N., Liu S. (2013). A cancer/testis antigen microarray to screen autoantibody biomarkers of non-small cell lung cancer. Cancer Lett..

[28] Middleton C.H., Irving W., Robertson J.F., Murray A., Parsy-Kowalska C.B., Macdonald I.K., McElveen J., Allen J., Healey G.F., Thomson B.J. (2014). Serum autoantibody measurement for the detection of hepatocellular carcinoma. PLoS ONE.

[29] Liu X.Y., Zheng C.B., Wang T., Xu J., Zhang M., Gou L.S., Jin L., Qi X., Zeng X., Li H. (2020). SPZ1 promotes deregulation of Bim to boost apoptosis resistance in colorectal cancer. Clin. Sci..

[30] Zhao M., Ye M., Zhou J., Zhu X. (2019). Prognostic values of transketolase family genes in ovarian cancer. Oncol. Lett..

[31] Li N., Zhan X. (2019). Identification of clinical trait-related lncRNA and mRNA biomarkers with weighted gene co-expression network analysis as useful tool for personalized medicine in ovarian cancer. EPMA J..

[32] Song M.H., Ha J.M., Shin D.H., Lee C.H., Old L., Lee S.Y. (2012). KP-CoT-23 (CCDC83) is a novel immunogenic cancer/testis antigen in colon cancer. Int. J. Oncol..

[33] Maheswaran E., Pedersen C.B., Ditzel H.J., Gjerstorff M.F. (2015). Lack of ADAM2, CALR3 and SAGE1 Cancer/Testis Antigen Expression in Lung and Breast Cancer. PLoS ONE.

