# Supplementary material for: Cancer-Testis Gene Biomarkers Discovered in Colon Cancer Patients
Source: Genes (Basel). 2022 May 1;13(5):807. doi: 10.3390/genes13050807 (PMC9141640; doi:10.3390/genes13050807)
Supplement: Supplementary file 1 [file genes-13-00807-s001.zip › genes-1689399-supplementary.pdf]

Table S1. Summary of the sequencing results for the desired genes.

Gene	Primer direction	Sequenced in					Sequence identity (%)
		Testis	NC	CC	CML or CLL	BC	
ADAM2	Forward	Yes	---	---	---	---	98.58
	Reverse	Yes	---	---	---	---	98.85
	Forward	---	---	3	---	---	0.00
	Reverse	---	---	3	---	---	0.00
	Forward	---	---	4	---	---	0.00
	Reverse	---	---	4	---	---	0.00
CTAG1A	Forward	Yes	---	---	---	---	98.96
	Reverse	Yes	---	---	---	---	99.49
	Forward	---	---	3	---	---	96.59
	Reverse	---	---	3	---	---	95.92
	Forward	---	---	14	---	---	100
ODF4	Forward	---	---	11	---	---	97.01
	Reverse	---	---	11	---	---	97.76
	Forward	---	---	14	---	---	97.74
	Reverse	---	---	14	---	---	99.24
LYZL6	Forward	Yes	---	---	---	---	98.70
	Reverse	Yes	---	---	---	---	97.80
	Forward	---	---	7	---	---	97.43
	Reverse	---	---	7	---	---	99.23
	Forward	---	---	8	---	---	98.04
	Reverse	---	---	8	---	---	98.38
SPZ1	Forward	---	---	11	---	---	96.85
	Reverse	---	---	11	---	---	99.55
TEX33	Forward	Yes	---	---	---	---	100
	Reverse	Yes	---	---	---	---	98.44
	Forward	---	---	24	---	---	0.00
	Reverse	---	---	24	---	---	0.00
TKTL2	Forward	Yes	---	---	---	---	99.85
	Reverse	Yes	---	---	---	---	99.71
	Forward	---	---	11	---	---	100
	Reverse	---	---	11	---	---	99.85
	Forward	---	---	14	---	---	99.85
	Forward	---	---	---	5	---	95.49
	Reverse	---	---	---	5	---	99.40
SCP2D1	Forward	---	---	11	---	---	99.69
	Reverse	---	---	11	---	---	99.38
	Forward	---	---	14	---	---	99.69
	Reverse	---	---	14	---	---	100
	Forward	---	---	---	1	---	99.06
	Reverse	---	---	---	1	---	99.07
	Forward	---	---	---	2	---	99.06
	Reverse	---	---	---	2	---	98.77
ACTRT1	Forward	Yes	---	---	---	---	99.45
	Reverse	Yes	---	---	---	---	99.43
	Forward	---	---	15	---	---	99.45
	Reverse	---	---	15	---	---	99.62
	Forward	---	14	---	---	---	92.81
	Reverse	---	14	---	---	---	94.48
	Forward	---	9	---	---	---	99.27
	Reverse	---	9	---	---	---	99.26
C10orf82	Forward	Yes	---	---	---	---	99.08
	Reverse	Yes	---	---	---	---	99.70

	Forward	---	---	---	---	5	85.03
	Reverse	---	---	---	---	5	91.00
	Forward	---	---	---	---	6	99.67
	Reverse	---	---	---	---	6	98.49
